# Pharmacological Inhibition of the Chemokine CXCL16 Diminishes Liver Macrophage Infiltration and Steatohepatitis in Chronic Hepatic Injury

**DOI:** 10.1371/journal.pone.0112327

**Published:** 2014-11-05

**Authors:** Alexander Wehr, Christer Baeck, Florian Ulmer, Nikolaus Gassler, Kanishka Hittatiya, Tom Luedde, Ulf Peter Neumann, Christian Trautwein, Frank Tacke

**Affiliations:** 1 Department of Medicine III, RWTH University-Hospital, Aachen, Germany; 2 Department of General, Visceral and Transplant Surgery, RWTH University-Hospital, Aachen, Germany; 3 Institute of Pathology, RWTH University-Hospital, Aachen, Germany; 4 Department of Pathology, Rheinische Friedrich-Wilhelms-University, Bonn, Germany; Bambino Gesu’ Children Hospital, Italy

## Abstract

Non-alcoholic fatty liver disease (NAFLD) is a major cause of morbidity and mortality in developed countries, resulting in steatohepatitis (NASH), fibrosis and eventually cirrhosis. Modulating inflammatory mediators such as chemokines may represent a novel therapeutic strategy for NAFLD. We recently demonstrated that the chemokine receptor CXCR6 promotes hepatic NKT cell accumulation, thereby controlling inflammation in experimental NAFLD. In this study, we first investigated human biopsies (n = 20), confirming that accumulation of inflammatory cells such as macrophages is a hallmark of progressive NAFLD. Moreover, CXCR6 gene expression correlated with the inflammatory activity (ALT levels) in human NAFLD. We then tested the hypothesis that pharmacological inhibition of CXCL16 might hold therapeutic potential in NAFLD, using mouse models of acute carbon tetrachloride (CCl_4_)- and chronic methionine-choline-deficient (MCD) diet-induced hepatic injury. Neutralizing CXCL16 by i.p. injection of anti-CXCL16 antibody inhibited the early intrahepatic NKT cell accumulation upon acute toxic injury *in vivo*. Weekly therapeutic anti-CXCL16 administrations during the last 3 weeks of 6 weeks MCD diet significantly decreased the infiltration of inflammatory macrophages into the liver and intrahepatic levels of inflammatory cytokines like TNF or MCP-1. Importantly, anti-CXCL16 treatment significantly reduced fatty liver degeneration upon MCD diet, as assessed by hepatic triglyceride levels, histological steatosis scoring and quantification of lipid droplets. Moreover, injured hepatocytes up-regulated CXCL16 expression, indicating that scavenging functions of CXCL16 might be additionally involved in the pathogenesis of NAFLD. Targeting CXCL16 might therefore represent a promising novel therapeutic approach for liver inflammation and steatohepatitis.

## Introduction

Non-alcoholic fatty liver disease (NAFLD) is defined as the accumulation of liver fat exceeding 5% of hepatocytes in the absence of significant alcohol intake (20 g/d for men, 10 g/d for women), viral infection, or any other specific etiology of liver disease. NAFLD has an increasing prevalence worldwide and is now the leading cause of liver diseases in Western countries [1]. The prevalence rate of NAFLD is reported to be 14–44% in the general population in Europe or the US and even 42.6–69.5% in people with type 2 diabetes [2], [3]. Patients with NAFLD, particularly those with non-alcoholic steatohepatitis (NASH), have a higher prevalence and incidence of clinically manifested cardiovascular disease as well and a 10-fold increased liver-related mortality owing to liver cirrhosis and hepatocellular carcinoma [3]. In a Danish study, after adjustment for sex, diabetes and cirrhosis at baseline, NAFLD-associated age-adjusted standardized mortality ratios (SMR) were 2.3 (95% CI 2.1–2.6) for all causes, 19.7 (95% CI 15.3–25.0) for hepatobiliary disease, and 2.1 (95% CI 1.8–2.5) for cardiovascular disease (CVD) [4]. Due to the lack of effective therapeutic measures and due to the epidemic of obesity and metabolic syndrome (affecting nearly 33% of the population in the US), NAFLD is projected to become the leading indication for liver transplantation in the next several years [5].

The progression of NAFLD, from hepatic lipid overload, steatosis, to non-alcoholic steatohepatitis (NASH) and to its complications liver fibrosis, cirrhosis or hepatocellular carcinoma, is causally linked to a massive inflammatory response in the liver [6]. However, despite the fact that the extent of hepatic inflammation is the predominant factor determining disease progression in NAFLD [7], no specific anti-inflammatory interventional approaches have entered clinical practice yet. There is a growing body of evidence that chemokines fulfill essential functions in regulating liver inflammation and NAFLD progression, thereby emerging as potential attractive targets for future therapeutic approaches [8].

Work from our laboratory has recently identified a yet unrecognized pathway promoting inflammation in experimental steatohepatitis in mice. Using mice either deficient for the chemokine receptor CXCR6 or transgenic for a fluorescent protein in one of the CXCR6 alleles, the accumulation of natural killer T (NKT) cells was identified as a rapid response to hepatocyte injury [9]. *Cxcr6^−/−^* mice lacking type-I NKT cells were protected against acute- and chronic liver failure in two independent models of liver fibrosis, showed significantly attenuated hepatic inflammation and reduced levels of pro-inflammatory cytokines like tumor necrosis factor (TNF), monocyte chemoattractant protein-1 (MCP-1) and interferon-γ (IFNγ). The adoptive transfer of wildtype (WT) NKT cells into *Cxcr6*-deficient mice in a model of steatohepatitis restored inflammation and liver fibrosis [9].

Our experimental data indicated that hepatic NKT cells specifically utilize its receptor CXCR6 for the rapid accumulation in injured livers, where they produce and release cytokines like interleukin 4 (IL-4) and IFNγ that act on macrophages (Kupffer cells) and possibly other hepatic cell compartments to initiate and perpetuate inflammation [9], [10]. We thus reasoned that inhibiting the cognate ligand for CXCR6, CXCL16, might hold therapeutic potential in steatohepatitis. Whereas in human inflamed livers CXCR6 is expressed by CD4 as well as CD8 T cells, NK cells and NKT cells the chemokine CXCL16 exists in a soluble and transmembrane form and is expressed by liver sinusoids, possibly allowing NKT cells to patrol hepatic vessels [11]–[13]. CXCL16 is also expressed by hepatic macrophages, likely favoring interactions between NKT and Kupffer cells [9]. Importantly, CXCL16 expression is up-regulated in acute or chronic liver injury in mice, but also in chronic liver diseases in humans [9]. In this study, we investigated the pharmacological inhibition of CXCL16 using a monoclonal antibody as a novel therapeutic approach in experimental steatohepatitis in mice.

## Materials and Methods

### Human liver samples

Liver biopsies of patients (n = 20) with NAFLD were collected and processed as described previously [14]. All liver biopsies were scored by an experienced pathologist, who was blinded to any experimental data, according to the NAS score [15]. Tissue from healthy patients (n = 3) with resections of hepatic hemangioma and normal ALT serum activity served as controls.

### Mice

C57bl/6 wild-type mice (B6-mice) were purchased from Janvier (Le Genest-Saint-Isle Saint-Berthevin Cedex, France) and were housed in a specific pathogen-free environment. All experiments were performed with male animals at 6 weeks of age under ethical conditions approved by the appropriate authorities according to German legal requirements.

### Induction of acute or chronic liver injury and anti-CXCL16 treatment

To induce acute liver injury, mice received 0.6 ml/kg body weight carbon tetrachloride (CCl_4_, Merck, Darmstadt, Germany) mixed with corn oil intraperitoneally and were sacrified 6 h thereafter. The control population of animals received the same volume of vehicle (corn oil) intraperitoneally. For induction of steatohepatitis and fibrosis, mice were fed with a methionine and choline deficient (MCD) diet for 6 weeks (MP Biomedicals, Cat.#390439, Solon, OH, USA) [9]. In order to block CXCL16, mice received 100 µg of monoclonal rat anti-mouse CXCL16 neutralizing antibody (R&D Systems, Clone #142417, Germany) intraperitoneally, whereas the control group received 100 µg of Bovine-Serum-Albumin (Sigma-Aldrich, St. Louis, MO, USA). Mice were either pre-treated 4 hours before acute CCl_4_ injury or received weekly injections during weeks 4–6 of a 6-week course of MCD diet. The i.p. route of antibody administration was chosen, because earlier studies had revealed a similar biodistribution in healthy as well as in diseased tissue compared to i.v. injections, and the i.p. administration was considered more reproducible in repetitive injections during the chronic injury model [16]–[19].

### Liver enzymes, histology and immunohistochemistry

Alanine (ALT) activities (UV test at 37°C) were measured from serum (Roche Modular preanalytics system, Rotkreuz Switzerland). Conventional H&E, Sirius red, and Ladewig staining were performed according to standard protocols [20]. Steatosis was scored by an experienced pathologist blinded to experimental data, as previously described [9], [15]. Liver sections from fixed paraffin blocks were immunohistochemically stained according to standard procedures using anti-mouse F4/80 and anti-human CD68 (Serotec), respectively [21]. CD68 and H&E pictures were analysed by quantifying the area fraction using imaging software in a blinded fashion (ImageJ).

### Isolation of primary hepatic cell populations

B6 mice were either treated with CCl_4_ or with corn oil three times per week for 2 weeks to induce liver fibrosis. Primary hepatocytes were isolated from murine livers by convential collagenase perfusion methodology as described before [22].

### Isolation of intrahepatic leukocytes

For the analysis of the intrahepatic leukocytes livers were perfused with 25 ml of phosphate buffered saline (PBS), minced with scissors and finally digested for 30 min with collagenase type IV (Worthington, Lakewood, NJ, USA) at 37°C. Digested extracts were pressed through 70 µm cell strainers. A small aliquot was stained with CD45 to assess the relative amount of intrahepatic leukocytes (CD45^+^) among all liver cells. The remaining liver cells were subjected to density gradient centrifugation (LSM-1077, PAA, Pasching, Austria) at 2000 rpm for 20 min at 25°C. Leukocytes were collected from the interface after centrifugation, washed twice with Hank’s balanced salt solution containing 2% BSA and 0.1 mM EDTA, and subjected to FACS [9].

### Flow cytometry

Six-color staining was conducted using combinations of following monoclonal antibodies: F4/80 (Serotec, Raleigh, NC, USA); CD4, CD11b (both eBioscience, San Diego, CA, USA); CD45, Gr1/Ly6C, Ly6G, CD19, NK1.1, CD8 and CD3 (all BD); CD1d tetramer loaded with αGalCer (ProImmune, Oxford, UK). Dead cells were excluded by Hoechst 33258 dye (Sigma-Aldrich, St. Louis, USA). Flow cytometric analysis was performed on a FACS-Canto (BD) and analysed with FlowJo (Tree Star, Ashland, OR, USA).

### Gene expression analysis

Liver tissue was shock-frozen in liquid nitrogen and stored at −80°C. RNA was purified from frozen liver samples by pegGOLD (peqLab, Erlangen, Germany), and cDNA was generated from 1 µg of RNA using a cDNA synthesis kit (Roche). Quantitative real-time PCR (qPCR) was performed using SYBR Green Reagent (Invitrogen, Carlsbad, CA, USA). Reactions were done twice in triplicate, and ß-actin values were used to normalize gene expression [9]. Primer sequences are available upon request.

### Hydroxyproline, cytokine and triglyceride measurements

The hepatic hydroxyproline content (reflecting total collagen) was measured as described before [23]. Tumor-necrosis-factor-α (TNFα) and monocyte chemoattractant protein-1 (MCP-1) were measured from protein extracts of liver by Elisa (eBioscience) [9]. Total protein content was quantified by photometric assay (Biorad, Hercules, Ca USA). The intrahepatic triglyceride content was measured by TG liquicolor mono (Human Diagnostics, Wiesbaden, Germany) according to the manufacturer’s instructions from homogenised snap-frozen liver samples.

### Statistical analysis

All experimental data are expressed as mean±SD. Differences between groups of mouse experiments were assessed by two-tailed unpaired Student t-test. Correlations between variables were asses by Pearson rank correlation test.

## Results

### Macrophage infiltration is a hallmark feature of steatohepatitis in humans

**Figure 1:**
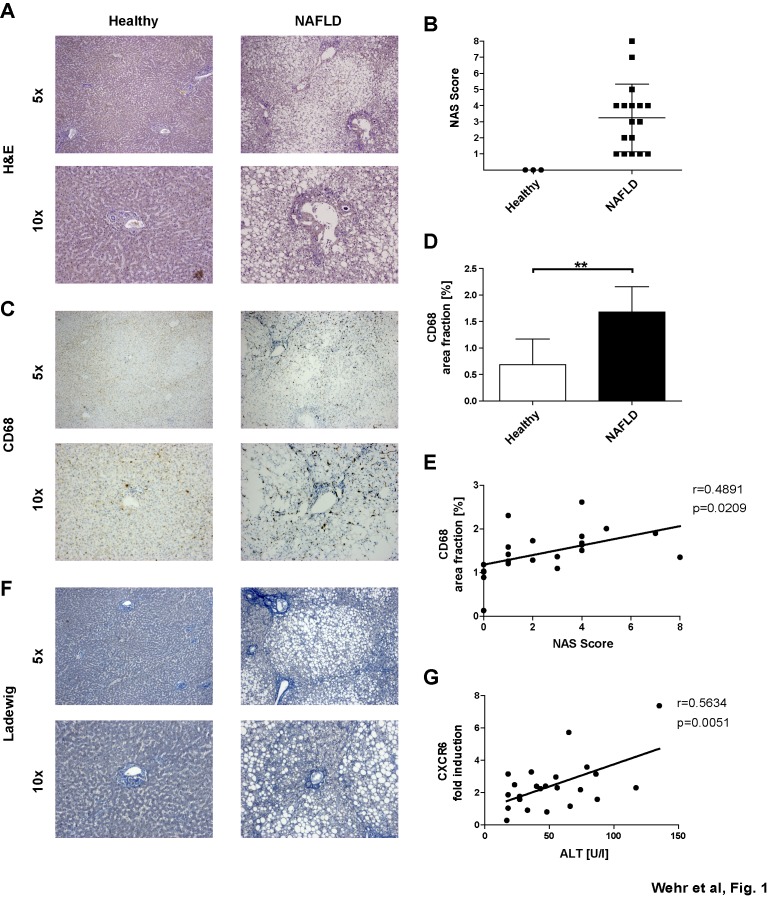
Hepatic macrophage infiltration is increased in liver of patients with non-alcoholic-steatohepatitis and accompanied by slightly enhanced CXCR6 expression. Liver biopsies of patients with NAFLD (n = 20) and healthy controls (n = 3) were analyzed. (**A–B**) Hematoxylin-Eosin stainings (H&E) of liver paraffin sections demonstrated steatosis development in patients with NAFLD compared to healthy controls. NAFLD severity was scored according to the NAS-Kleiner-Score. (**C–D**) CD68 immunohistochemical stainings showed a significant increase of intrahepatic macrophages in livers of NAFLD patients compared to healthy controls. (**E**) Hepatic macrophages (CD68 positive) significantly correlated with the NAS. (**F**) Patients with NAFLD developed fibrosis, as evidenced by collagen deposition (blue) in Ladewig staining of liver biopsies. (**G**) High CXCR6 expression correlated with elevated serum ALT levels. P-values and correlation coefficients are given in the figure. All data are expressed as mean ± SD. *p<0.05, **p<0.005, ***p<0.001.

Experimental rodent models of fatty liver diseases revealed that intrahepatic macrophages massively increase in response to hepatic lipid overload and promote the progression of liver fibrosis as an aberrant wound-healing response [6]. We examined liver biopsies of 10 patients with NAFLD undergoing bariatric surgery in comparison to healthy liver tissue obtained from patients undergoing resections of benign liver lesions (i.e. hemangioma). NAFLD was characterized by steatosis, hepatocyte ballooning and a pronounced infiltrate of mononuclear cells (Fig. 1A), as reflected by the NAFLD Activity Score (NAS) (Fig. 1B). Upon immunohistochemical staining for the macrophage marker CD68, the accumulation of macrophages, especially around portal fields, was identified as a hallmark feature of NAFLD (Fig. 1C–D), in line with current literature [24]. Additionally, the hepatic macrophage content as quantified by CD68 immunohistochemistry significantly correlated with the NAS (Fig. 1E). In some of the patients, NAFLD was associated with moderate to severe hepatic fibrosis, characterized by extracellular matrix deposition around and partially bridging between portal fields (Fig. 1F).

Following our hypothesis that the CXCR6-dependent recruitment of NKT cells might contribute to enhanced macrophage accumulation in NAFLD, we assessed hepatic gene expression levels for CXCR6 by quantitative PCR. Hepatic CXCR6 expression showed a significant correlation with elevated serum ALT levels in patients suffering from NAFLD (Fig. 1G). These data prompted the current study to address on a functional level in mouse models whether inhibition of the CXCL16-CXCR6 pathway might be suitable to inhibit steatohepatitis and macrophage accumulation in NAFLD.

### Anti-CXCL16 antibody sufficiently blocks early hepatic NKT cell accumulation upon acute liver injury

**Figure 2:**
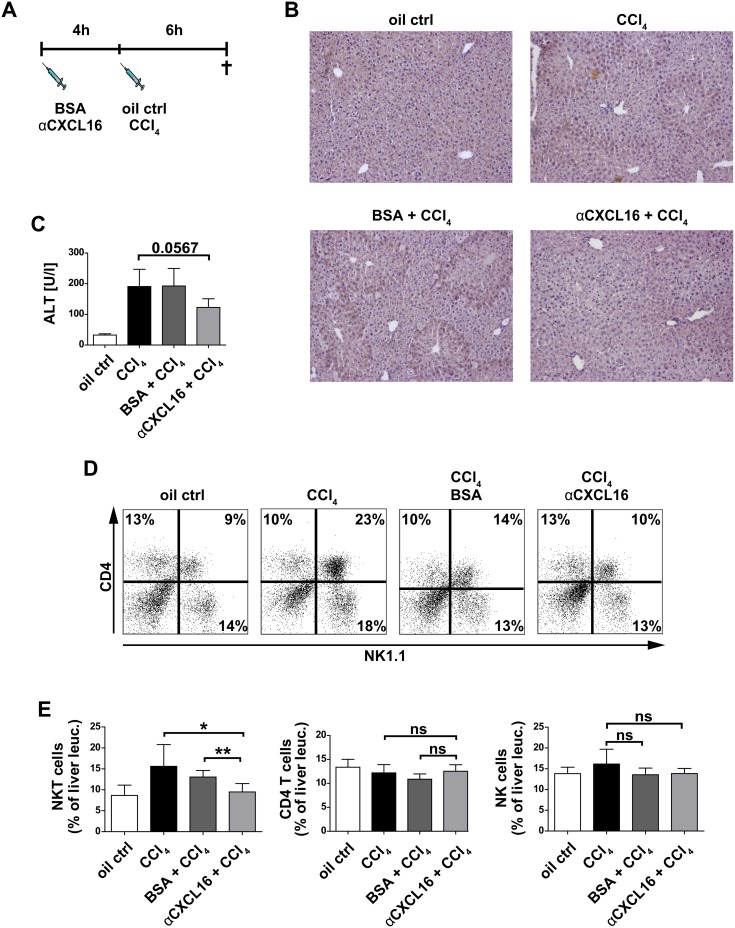
Anti-CXCL16 antibody sufficiently blocks early hepatic NKT cell accumulation upon acute liver injury. (**A**) B6-mice received a singular intraperitoneal application of either αCXCL16 or BSA (unspecific protein control) 4 hours before injection of carbon tetrachloride (CCl_4_). Mice were sacrificed 6 hours after CCl_4_ application. (**B–C**) As evidenced by H&E stainings and serum ALT activity, αCXCL16 treated mice showed reduced toxic liver damage *in vivo* compared to BSA treated mice. (**D–E**) Flow cytometric analysis of intrahepatic leukocytes (representative FACS plots in D, statistical analyses in E) showed a significant decrease of hepatic NKT cells in mice that received αCXCL16 compared to controls. Other CXCR6 expressing cells like CD4 T- and NK cells were not altered with respect to their migration behavior into the injured liver upon αCXCL16 injection. All data are expressed as mean ± SD from three independent experiments, summarizing n = 6 animals per group. *p<0.05, **p<0.005, ***p<0.001.

Our prior experiments, using intravital multiphoton-imaging of *Cxcr6^+/gfp^* transgenic mice and FACS phenotyping of CXCR6^+^ immune cells, indicated that hepatic NKT cells accumulate very early after injury - e.g., at a maximum as early as 6 hours after injection of the hepatotoxin CCl_4_ - in the liver in a CXCR6-dependent manner [9]. We therefore hypothesized that inhibition of CXCL16, the specific ligand for CXCR6, could efficiently block this early NKT-dependent step in the initiation of the hepatic inflammatory response. Therefore, c57bl/6 wildtype mice were treated with anti-CXCL16 antibody (αCXCL16) or Bovine-Serum-Albumin (BSA) as control i.p., followed by CCl_4_ injection 4 hours later (Fig. 2A). Six hours after CCl_4_, the liver showed initial signs of injury with cellular swelling, some necrosis and inflammatory cell infiltration in histology (Fig. 2A+B). In the CCl_4_ injury model, massive necrosis of hepatocytes and even further inflammatory infiltration peaks at around 24 hours after injury (Fig. S1A) [23]. Serum ALT levels are mildly elevated at 6 hours as a measure for early acute liver injury (Fig. 2C). Notably, liver injury appeared slightly attenuated in αCXCL16-treated mice at this early time-point (Fig. 2B–C), as indicated by moderately reduced ALT levels in αCXCL16-treated mice.

To investigate the infiltrating immune cells, intrahepatic leukocytes were phenotypically characterized by FACS at 6 hours after CCl_4_, and NKT cells were identified as CD45^+^CD4^+^NK1.1^+^ cells. Interestingly, the early NKT cell accumulation could be efficiently blocked by administration of αCXCL16, as hepatic NKT cell numbers were reduced to the level of non-injured control mice due to αCXCL16 treatment (Fig. 2D–E). Mice that received BSA did not show the altered hepatic NKT cell accumulation, thereby excluding an unspecific protein reaction. Moreover, the effect of αCXCL16 treatment appeared specific to NKT cells, as other lymphocytes, which also express the chemokine receptor CXCR6 like CD4 and NK cells [9], were not affected by the antibody injection.

### Therapeutic administration of αCXCL16 inhibits macrophage infiltration and hepatic inflammation in experimental steatohepatitis

**Figure 3:**
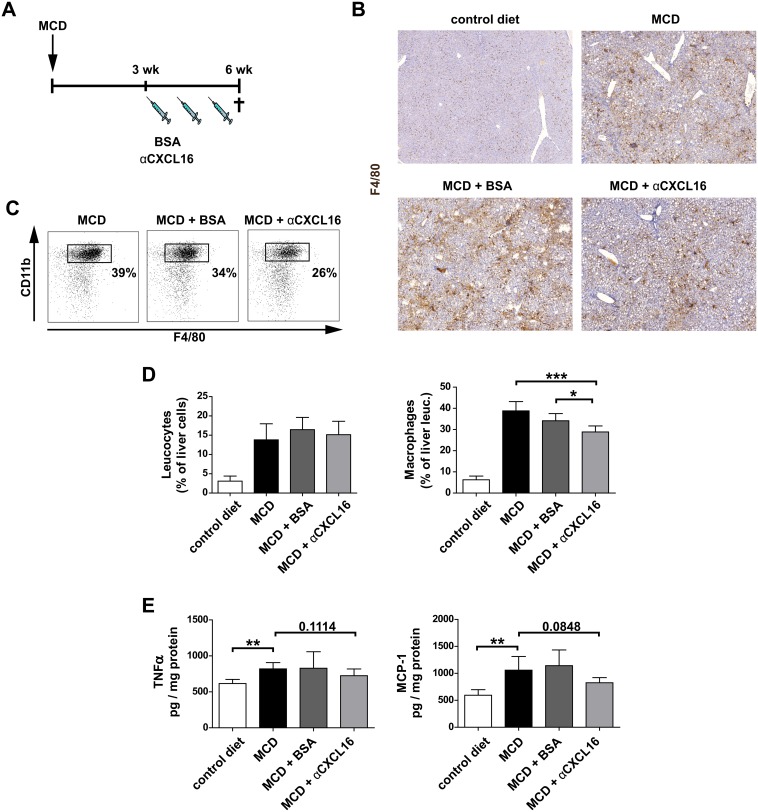
Therapeutic administration of αCXCL16 inhibits macrophage infiltration and hepatic inflammation in experimental steatohepatitis. (**A**) B6-mice were subjected to a methionine-choline deficient (MCD) diet over 6 weeks. During the last three weeks of progressing steatohepatitis, mice were treated with αCXCL16 or BSA i.p. once per week. (**B–D**) F4/80 immunohistochemical staining of liver tissue (B) and flow cytometric analysis of intrahepatic leukocytes (representative plots, C; statistical analysis, D) showed a significant reduction of infiltrating CD11b^+^F4/80^+^ macrophages in αCXCL16 treated mice compared to controls. (**E**) Pro-inflammatory cytokines like TNFα and MCP-1 were slightly decreased in liver tissue of mice that received weekly αCXCL16 injections compared to controls during MCD diet. All data are expressed as mean ± SD from three independent experiments, summarizing n = 6 animals per group. *p<0.05, **p<0.005, ***p<0.001.

Our experiments indicated that the administration of αCXCL16 effectively blocked the early accumulation of hepatic NKT cells in response to hepatocyte injury, thus possibly allowing to dampen the subsequent inflammatory response in the liver. In order to test this, B6-mice were subjected to a methionine-choline deficient (MCD) diet over 6 weeks of time. The lack of the amino acids methionine and choline leads to hepatic inflammation induced by accumulation of fatty acids and pro-inflammatory immune cells [25]. During the last three weeks of progressing steatohepatitis in a 6 weeks course of MCD diet, mice were treated with 100 µg αCXCL16 or BSA i.p. once per week (Fig. 3A). In line with our hypothesis, αCXCL16-treated mice showed a significant reduction of pro-inflammatory macrophages as evidenced by immunohistological stainings for F4/80 and FACS analysis of intrahepatic leukocytes (Fig. 3B–D). Of note, hepatic NKT cell numbers were very low in all groups fed with MCD diet (data not shown), due to the rapid activation induced cell death (AICD) of NKT in hepatic injury [9], [26]. The strong reduction of hepatic macrophages upon αCXCL16 administration during the MCD diet was accompanied by a trend towards reduced intrahepatic levels of pro-inflammatory cytokines like TNFα and MCP-1 (Fig. 3E).

### Therapeutic administration of αCXCL16 attenuates steatosis development in experimental steatohepatitis

**Figure 4:**
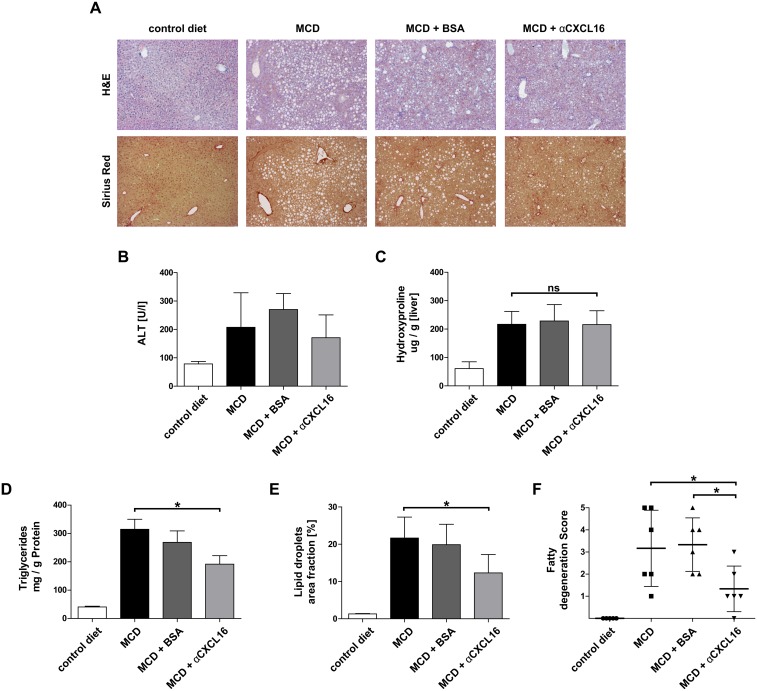
Therapeutic administration of αCXCL16 attenuates steatosis development in experimental steatohepatitis. (**A**) Liver histology showed no altered fibrosis development, but a clear reduction of lipid accumulation in injured livers of αCXCL16 treated mice compared to controls as evidenced by H&E- and Sirius red stainings. (**B–C**) Serum ALT activity and hydroxyproline content were not affected by the administration of αCXCL16. (**D–E**) Intrahepatic triglyceride content and periportal lipid droplets were significant reduced in mice that received weekly αCXCL16 injections compared to controls during MCD diet. (**F**) Fatty degeneration score was significantly decreased in αCXCL16 treated mice compared to controls. All data are expressed as mean ± SD from three independent experiments, summarizing n = 6 animals per group. *p<0.05, **p<0.005, ***p<0.001.

We next analyzed whether pharmacological inhibition of CXCL16 might represent a successful therapeutic approach to limit disease progression in experimental steatohepatitis. Treatment with αCXCL16 (experimental design as in Fig. 3A) did not significantly affect hepatic fibrosis in the MCD-induced chronic liver injury, as assessed by collagen deposition in Sirius red histological stainings (Fig. 4A), by serum ALT levels (Fig. 4B) or by quantification of the hepatic hydroxyproline content (Fig. 4C) as a sensitive measure for collagen fibers in the liver. However, already by conventional H&E stainings from liver histology, it was apparent that αCXCL16 administration not only reduced the inflammatory infiltrate, but also the numbers of lipid droplets in hepatocytes (Fig. 4A). Measurement of the hepatic triglyceride content as well as histological quantification of lipid droplets confirmed significantly attenuated steatosis development, if MCD-fed mice received αCXCL16 during the last 3 weeks of a 6-week MCD diet-induced metabolic injury (Fig. 4D–E). In agreement with these data, the fatty degeneration score was also significant decreased in mice treated with αCXCL16 (Fig. 4F).

## Discussion

Non-alcoholic fatty liver disease is a raising health burden in Western countries, which affect about 30% of the general adult population, is associated with an increased mortality and promote several medical complications like hepatocarcinogenesis in humans [3], [27]. Within the liver, hepatocyte apoptosis, ER stress and oxidative stress are key contributors to hepatocellular injury. Moreover, lipotoxic mediators and danger signals activate the hepatic macrophage pool, mainly Kupffer cells, which are critical for the initiation and perpetuation of the inflammatory response and release several inflammatory mediators [6]. Such mediators, mainly cytokines and chemokines, attract inflammatory cells and activate parenchymal as well as non-parenchymal liver cells during NAFLD progression [21]. In our study, we provide experimental evidence that interfering with the CXCR6-CXCL16 pathway might hold therapeutic potential in NAFLD. The administration of an anti-CXCL16 antibody blocked the rapid accumulation of patrolling hepatic NKT cells in response to acute hepatocyte injury. In line, therapeutic administration of this antibody in experimental chronic metabolic injury attenuated hepatic macrophage infiltration, pro-inflammatory cytokine levels and steatosis development in mice.

On the one hand, our experiments using αCXCL16 revealed that blocking this chemokine pathway almost completely abolished the rapid accumulation of hepatic NKT cells in response to an acute injury. This is well in line with a prior *in vitro* experiment from our group, demonstrating that CXCR6 is specifically required by NKT, but not other CXCR6-expressing lymphocytes, to migrate towards CXCL16 [9]. Moreover, it had been reported that CXCL16 neutralization reduced accumulation of mature NK1.1^+^, but not immature NK1.1^−^ NKT cell recent thymic emigrants in the liver in homeostatic conditions *in vivo* [28]. NKT cells display a unique population of unconventional T cells that express both a T cell receptor (Vα-14-Jα18 in mice; Vα24-Jα18 in humans) and NK1.1 receptor from NK cells [10], [29]. There are different types of NKT cell subsets that are defined by the ability of recognizing α-galactosylceramide (α-GalCeramide) presented by the non-classical MHC-like molecule CD1d [10], termed type-I classical, type-II non classical and CD1d-independent NK1.1^+^ NKT cells. Especially the type-I NKT cells, which the by far largest subset in the liver, have the ability to secrete various types of cytokines within a very short time period after activation, including IL-4 and IFNγ [9], [10]. In line, mice that are deficient for the CXCL16 chemokine displayed a reduced number of liver NKT cells, decreased production of IFNγ and IL-4 by administration of α-GalCeramide and impaired (Th1-type) inflammatory responses against *Propionibacterium acnes*-infections *in vivo* [30].

On the other hand, our experiments indicate that αCXCL16 could be an interesting therapeutic strategy in hepatic inflammation and steatohepatitis. Importantly, the same αCXCL16 antibody had been tested as an interventional approach for severe inflammatory conditions before. In a murine immunological liver injury induced by Bacille Calmette-Guerin and lipopolysaccharide, mice treated with αCXCL16 showed reduced liver injury, inflammation and improved survival [31]. Administration of αCXCL16 also reduced colonic inflammation in mouse models of on dextran sodium sulfate- and trinitrobenzene sulfonic acid-induced colitis [32]. In our hands, therapeutic administration of αCXCL16 during the last three weeks of a 6-weeks course of MCD diet in mice significantly reduced the number of hepatic macrophages, alongside minor reductions in intrahepatic levels of pro-inflammatory cytokines, and steatosis development. The link between macrophages and progression of fatty liver degeneration is well established, as macrophages release many inflammatory mediators that not only attract additional immune cells, but also drive oxidative stress and intrahepatocytic lipid accumulation [6], [25], [33]. Our experiments now indicate that blocking CXCL16 effectively reduces pro-inflammatory macrophages in experimental steatohepatitis. As macrophages do not express CXCR6 [9] (and data not shown), the effects of αCXCL16 on hepatic macrophages are likely the result on inhibiting NKT cell accumulation early in the course of injury. In fact, reducing the additional pro-inflammatory effects of NKT cells in injured liver was shown before to significantly ameliorate the extent of chronic hepatic inflammation and macrophage activation [9], [26], [34]–[38]. Our study now emphasizes that this can be sufficiently achieved by targeting the CXCR6/CXCL16 chemokine axis.

However, an alternative explanation for the effects of inhibiting CXCL16 needs to be considered. It has been shown previously that CXCL16 can act as a scavenging receptor for oxidized low density lipoprotein (oxLDL) [39]–[41]. In order to explore whether CXCL16 might act as a scavenger for lipids or lipid-protein complexes on hepatocytes in conditions of NAFLD, we have isolated hepatocytes from normal and from chronically injured B6 mice. In fact, CXCL16 gene expression was about 7-fold up-regulated in hepatocytes from injured mice (Fig. S1B). Thus, it is possible that inhibiting CXCL16 by an antibody not only affects immune cell migration and function, but has additional effects on the lipid accumulation in hepatocytes. Further studies are needed to investigate the relevance of scavenging functions by CXCL16 for NAFLD progression.

Importantly, although steatosis development was attenuated upon αCXCL16 in the MCD diet model, hepatic fibrosis progression was not significantly affected. Several factors might explain this observation. First, we have chosen a relatively low dose of the antibody (100 µg) and only three injections (once per week); in experimental models of murine colitis, mice were subjected to 500 µg of αCXCL16 i.p. every day for the total of 7 days [32]. Thus, different doses and more frequent applications should be considered for future studies. Second, the MCD diet model is characterized by a rather scattered deposition of collagen in the liver and a rather mild fibrosis, especially when mice are analyzed after 6 weeks of diet [25]. Thus, in order to address the possible impact of αCXCL16 on fibrosis, ‘classical’ fibrosis models, modifications of the metabolic injury model or mice with a more fibrosis-prone genetic background (e.g., Balb/c instead of B6) should be investigated [33], [42]. Third, it is currently unclear to which extent infiltrating vs. resident hepatic macrophages contribute to progression of liver fibrosis [43]. Based on the flow cytometric characterization in our study, αCXCL16 primarily affected the CD11b^+^F4/80^+^ macrophage population, which is mainly derived from infiltrating inflammatory Ly-6C^+^ (Gr1^+^) monocytes [23]. As shown before by selectively blocking Ly-6C^+^ monocyte influx in chronic liver injury models, this macrophage subset appeared functionally essential for steatohepatitis, but less relevant for fibrosis progression [25]. In line, a recent study demonstrated a crucial role of inflammatory CD11b^+^ macrophages in CCl_4_ induced acute liver injury. The hepatic injury was driven by macrophage-derived TNFα and Fas-Ligand (FasL), as inhibition of TNFα and FasL dampened the hepatic injury induced by a single CCl_4_ injection. Interestingly, CD1d-deficient mice lacking both type I and II NKT cells, did not show significant differences in the progression of acute liver injury compared with control mice in this study [44]. However, this study was conducted in Balb/c mice. A similar study with CD1d-deficient mice on a c57bl/6 background showed that NKT-cell-deficient mice were protected from acute toxic liver injury [35], in well agreement with our previous [9] and the current study. Possibly, these data might indicate that hepatic NKT cells are especially relevant in Th1-prone inflammatory conditions.

Taken together, our study provides experimental evidence that targeting the chemokine CXCL16 is a promising strategy in fatty liver diseases. The inhibition of CXCR6-dependent NKT cell accumulation as an important inflammatory cell component ameliorated the extent of hepatic inflammation, macrophage activation and steatosis development in experimental metabolic injury. Future studies should address the optimal dose and administration schedule and should aim at translating these findings into novel approaches for human NAFLD.

## Supporting Information

Figure S1 **(A) Kinetics of necrosis development in CCl_4_ injured B6 mice.** Six hours after CCl_4_ injection, the liver showed initial signs of injury with cellular swelling, some necrosis and inflammatory cell infiltration. Massive necrosis of hepatocytes and even further inflammatory infiltration peaks at around 24 hours after injury. **(B) CXCL16 mRNA expression in primary hepatocytes.** CXCL16 is highly up-regulated in primary hepatocytes isolated from chronically injured mouse livers compared to primary hepatocytes from control livers.
(TIF)
