# Supplementary figures and images for: Pharmacological Inhibition of the Chemokine CXCL16 Diminishes Liver Macrophage Infiltration and Steatohepatitis in Chronic Hepatic Injury
Source: PLoS One. 2014 Nov 5;9(11):e112327. doi: 10.1371/journal.pone.0112327 (PMC4221470; doi:10.1371/journal.pone.0112327)

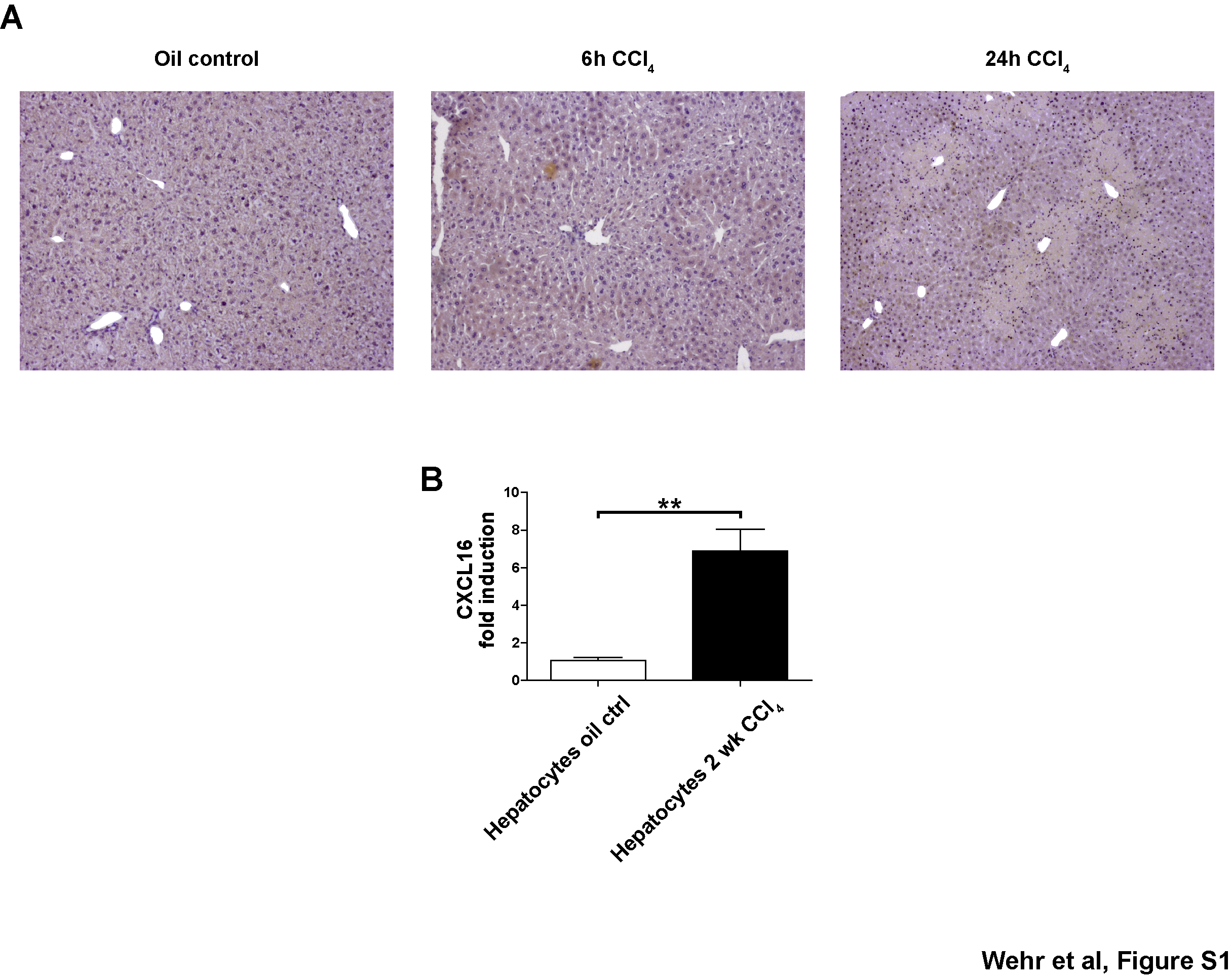

Supplement: Figure S1 — (A) Kinetics of necrosis development in CCl4 injured B6 mice. Six hours after CCl4 injection, the liver showed initial signs of injury with cellular swelling, some necrosis and inflammatory cell infiltration. Massive necrosis of hepatocytes and even further inflammatory infiltration peaks at around 24 hours after injury. (B) CXCL16 mRNA expression in primary hepatocytes. CXCL16 is highly up-regulated in primary hepatocytes isolated from chronically injured mouse livers compared to primary hepatocytes from control livers. (TIF) [file pone.0112327.s001.tif]
